# Epigenetic regulation of NOTCH1 and NOTCH3 by KMT2A inhibits glioma proliferation

**DOI:** 10.18632/oncotarget.18668

**Published:** 2017-06-27

**Authors:** Yin-Cheng Huang, Sheng-Jia Lin, Hung-Yu Shih, Chung-Han Chou, Hsiao-Han Chu, Ching-Chi Chiu, Chiou-Hwa Yuh, Tu-Hsueh Yeh, Yi-Chuan Cheng

**Affiliations:** ^1^ College of Medicine, Chang Gung University, Taoyuan, Taiwan; ^2^ Department of Neurosurgery, Chang Gung Memorial Hospital at Linkou Medical Center, Taoyuan, Taiwan; ^3^ Graduate Institute of Biomedical Sciences, College of Medicine, Chang-Gung University, Taoyuan, Taiwan; ^4^ School of Medicine, College of Medicine, Chang-Gung University, Taoyuan, Taiwan; ^5^ Neuroscience Research Center, Chang Gung Memorial Hospital at Linkou Medical Center, Taoyuan, Taiwan; ^6^ Institute of Molecular and Genomic Medicine, National Health Research Institutes, Zhunan, Taiwan; ^7^ Department of Neurology, Taipei Medical University Hospital, Taipei, Taiwan; ^8^ School of Medicine, Taipei Medical University, Taipei, Taiwan

**Keywords:** KMT2A, NOTCH, glioma, zebrafish

## Abstract

Glioblastomas are among the most fatal brain tumors; however, the molecular determinants of their tumorigenic behavior are not adequately defined. In this study, we analyzed the role of KMT2A in the glioblastoma cell line U-87 MG. KMT2A knockdown promoted cell proliferation. Moreover, it increased the DNA methylation of *NOTCH1* and *NOTCH3* and reduced the expression of *NOTCH1* and *NOTCH3*. NOTCH1 or NOTCH3 activation inhibited U-87 MG cell proliferation, whereas NOTCH1 and NOTCH3 inhibition by shRNAs induced cell proliferation, thus demonstrating the tumor-suppressive ability of NOTCH1 and NOTCH3 in U-87 MG cells. The induced cell proliferation caused by KMT2A knockdown could be nullified by using either constitutively active NOTCH1 or constitutively active NOTCH3. This result demonstrates that KMT2A positively regulates NOTCH1 and NOTCH3 and that this mechanism is essential for inhibiting the U-87 MG cell proliferation. The role of KMT2A knockdown in promoting tumor growth was further confirmed *in vivo* by transplanting U-87 MG cells into the brains of zebrafish larvae. In conclusion, we identified KMT2A-NOTCH as a negative regulatory cascade for glioblastoma cell proliferation, and this result provides important information for KMT2A- or NOTCH-targeted therapeutic strategies for brain tumors.

## INTRODUCTION

Lysine (K)-specific methyltransferase 2A [*KMT2A*, also known as mixed lineage leukemia 1 (*MLL1*)] encodes a transcriptional coactivator that regulates gene expression during early development and hematopoiesis [1]. Multiple chromosomal translocations involving this gene have been identified in certain acute lymphoid leukemias and acute myeloid leukemias [2]. The encoded protein contains multiple conserved functional domains. One of these domains, SET [Su(var), Enhancer of zeste, trx], is responsible for histone H3 lysine 4 (H3K4) methyltransferase activity, which mediates chromatin modifications associated with epigenetic transcriptional activation. KMT2A is processed by the enzyme Taspase 1 into two fragments, MLL^N^ and MLL^C^. These fragments reassociate and further assemble into different multiprotein complexes that regulate the transcription of specific target genes including many *HOX* genes [3]. In addition to H3K4 methyltransferase activity, KMT2A regulates DNA methylation. The CXXC domain in KMT2A binds to unmethylated CpG islands and protects CpG clusters within target genes such as *HOXA9* from methylation [4–6]. KMT2A knockdown reverses the methylation protection status in the previously protected CpG clusters of *HOXA9* [4].

KMT2A also plays an important role in maintaining specific gene expression for neurogenesis, hematopoiesis, and osteogenesis [7–9]. Conditional *KMT2A* knockout regulates the survival, proliferation, and differentiation of subventricular zone neural stem cells in postnatal mouse brains [7]. In zebrafish embryos, disrupting *Kmt2a* expression by using morpholino antisense oligonucleotides and a dominant-negative variant resulted in the downregulated proliferation of neural progenitors, premature differentiation of neurons, and impaired gliogenesis [9]. However, the role of KMT2A and its downstream signaling in brain tumors, particularly the role of epigenetic regulatory activity, remains to be clarified.

Genes belonging to the *KMT2* family have been implicated in many mammalian cancers [10], and mutations in these genes are among the most frequent alterations in human cancer [11]. In hematopoietic cells, KMT2A translocations result in oncogenic fusion proteins that recruit DOT1-like histone H3K79 methyltransferase, which changes the epigenetic identity of the cells and drives a subset of infantile and adult leukemias [10, 12]. These studies have demonstrated the oncogenic character of KMT2A. However, the role of *KMT2A* in regulating tumor progression remains unclear, because most studies to date have focused on the function of the translocated *KMT2A* gene and the role of the fusion proteins resulting from translocation. Heddleston et al. demonstrated high expression levels of KMT2A in glioma stem cells (GSCs) [13]. KMT2A knockdown inhibited the expression of hypoxic-inducible factors and the vascular endothelial growth factor. In addition, KMT2A depletion reduced the self-renewal ability of GSCs and tumorigenicity [13, 14]. These studies have indicated that KMT2A is associated with glial-derived tumors.

In the current study, we examined the role of KMT2A in the U-87 MG glioblastoma cell line. We demonstrated that KMT2A is essential for the inhibition of tumor cell proliferation by using both *in vitro* and *in vivo* models. We further demonstrated that KMT2A knockdown increased *NOTCH1* and *NOTCH3* methylation, and the KMT2A-NOTCH1/3 cascade negatively regulated U-87 MG cell proliferation. Our results revealed the tumor-suppressive character of KMT2A, NOTCH1, and NOTCH3 in U-87 MG cells.

## RESULTS

### KMT2A knockdown induces glioma cell proliferation

**Figure 1:**
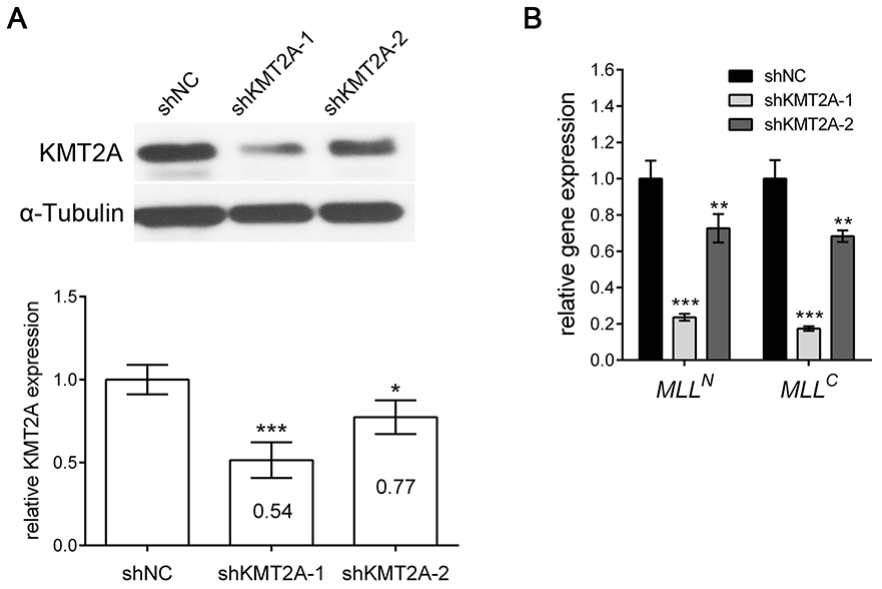
*shKMT2A* effectively downregulates KMT2A expression *shKMT2A* downregulates the expression of *KMT2A* in U-87 MG cells. **(A)** Western blot analysis indicated that KMT2A expression was inhibited by *shKMT2A*-1 and *shKMT2A*-2, and the result was quantified. *shKMT2A-1* was more efficient than *shKMT2A-2*. **(B)** RT-PCR demonstrated that *shKMT2A* inhibits the expression of *MLL^N^* and *MLL^C^*. *, p < 0.05; **, p < 0.01; ***, p < 0.001.

We investigated the role of KMT2A in U-87 MG cells (grade IV glioma cell line). We designed two shRNAs specifically targeting KMT2A (*shKMT2A-1* and *shKMT2A-2*) and confirmed the effectiveness of KMT2A shRNAs through real-time RT-PCR and Western blot analysis. Cells transfected with *shKMT2A* exhibited significantly reduced KMT2A expression (Figure 1A). KMT2A is normally cleaved at two conserved sites, generating N-terminal p320 (*MLL^N^*) and C-terminal p180 (*MLL^C^*) fragments, which form a stable complex that localizes to subnucleus compartments [15]. Accordingly, we examined whether *shKMT2A* could affect these two fragments. The result reveals that *shKMT2A* downregulated the mRNA expression of both the N-form (*MLL^N^*) and C-form (*MLL^C^*) of *KMT2A* (Figure 1B).

**Figure 2:**
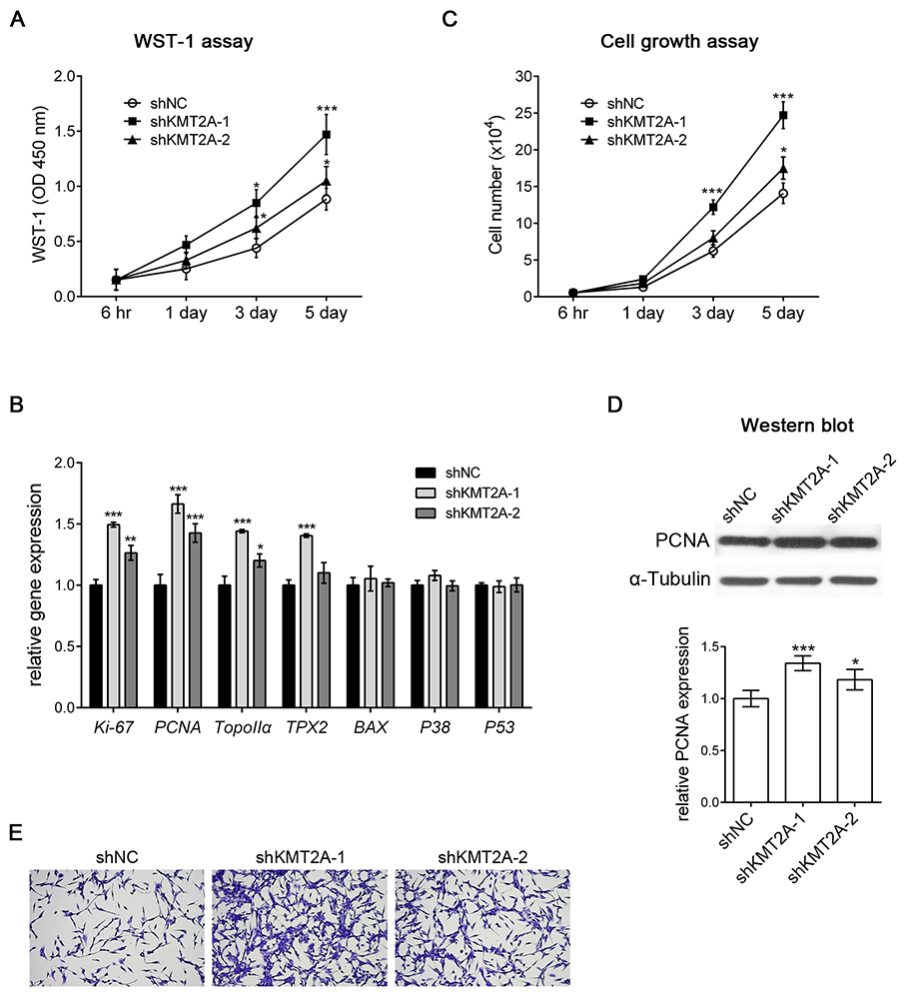
*shKMT2A* induces U-87 MG cell proliferation **(A)** The effect of shKMT2A was examined using a WST-1 assay, which demonstrated that *shKMT2A* induces U-87 MG cell proliferation. **(B)** Cell proliferation and survival markers were analyzed using real-time RT-PCR. *shKMT2A* induces proliferation markers [Ki-67, PCNA, Topoisomerase IIα (TopoIIα), and TPX2] without a significant alteration of apoptosis markers (BAX, P38 MAPK, and P53). **(C)** Cell growth assay showing *shKMT2A* induces U-87 MG cell growth. **(D)** Western blot analysis indicated that *shKMT2A* upregulates the expression level of PCNA. **(E)** Representative images revealing that *shKMT2A* induces the growth of U-87 MG cells. Cells were stained with crystal violet. *, p < 0.05; **, p < 0.01; ***, p < 0.001.

We next examined the effect of KMT2A knockdown in U-87 MG cells by using a WST-1 assay, and the result revealed that *shKMT2A* significantly induced U-87 cell proliferation (Figure 2A), indicating that KMT2A downregulation was essential for U-87 cell proliferation. We further analyzed cell proliferation and survival markers by using real-time RT-PCR, and the result demonstrated that the mRNA expression levels of the proliferation markers *Ki-67*, *PCNA*, *Topoisomerase IIα* (*TopoIIα*), and *TPX2* were upregulated in KMT2A knockdown cells. By contrast, the apoptosis-related markers *BAX*, *P38 MAPK*, and *P53* were unaffected by KMT2A knockdown (Figure 2B). To confirm the effect of *shKMT2A* on cell proliferation, the number of cells grown was measured using a cell growth assay (Figure 2C), and the proliferating cells were detected by proliferating cell nuclear antigen (PCNA), which was examined through Western blotting (Figure 2D). This result confirms that KMT2A is essential for inhibiting U-87 MG cell proliferation without affecting apoptosis.

### KMT2A upregulates the expression of NOTCH receptors through methylation

**Figure 3:**
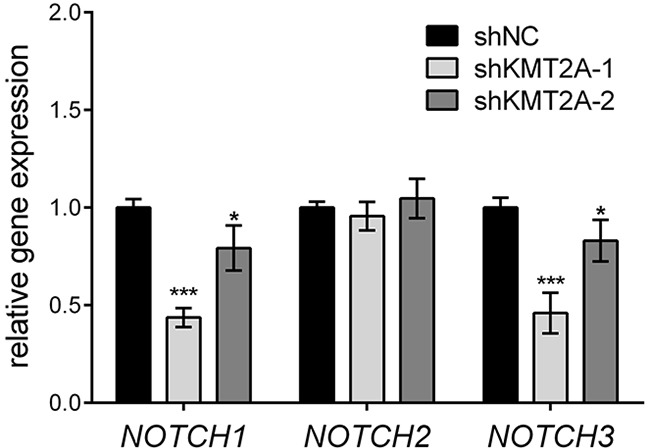
KMT2A upregulates NOTCH signaling The expression of *NOTCH* genes was examined using real-time PCR. In KMT2A-deficient U-87 MG cells, the expression levels of *NOTCH1* and *NOTCH3* were downregulated, whereas the expression level of *NOTCH2* was unaffected when compared with the control. *, p < 0.05; ***, p < 0.001.

The *Drosophila* homologue of KMT2A, Trithorax (Trx), was demonstrated to collaborate with Notch in gene activation [16]. Mutations in H3K27me3 demethylase Utx, another member of the Trithorax Group (TrxG) of proteins, induce Notch signaling in Drosophila [17], suggesting that the Notch signaling pathway is regulated by KMT2A. Accordingly, we examined the expression of NOTCH receptors by using real-time RT-PCR. KMT2A knockdown significantly reduced the expression of *NOTCH1* and *NOTCH3*, but it did not affect the expression of *NOTCH2* (Figure 3). This result indicates that KMT2A is a positive regulator of *NOTCH1* and *NOTCH3* transcription, thus suggesting that KMT2A selectively mediates NOTCH signaling.

**Figure 4:**
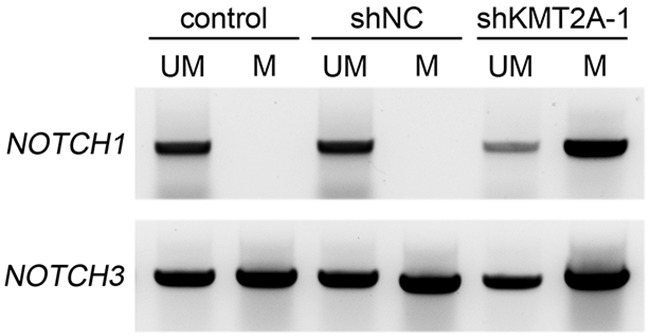
Methylation-specific PCR reveals *NOTCH1* and *NOTCH3* methylation patterns in U-87 MG cell line M, primers specific for methylated DNA; UM, primers specific for unmethylated DNA. Signals were observed only in unmethylated *NOTCH1*, indicating that both alleles were unmethylated. By contrast, positive signals were observed in both methylated and unmethylated *NOTCH3*, indicating that only one allele was methylated. The signaling observed for methylated NOTCH1 and NOTCH3 significantly increased upon KMT2A knockdown. This experiment was performed using an EZ DNA Methylation-Lightning Kit (Zymo Research). ***, p < 0.001.

The CXXC domain in KMT2A binds to unmethylated CpG islands and protects CpG clusters within target genes from methylation [4–6]. CpG DNA methylation of *NOTCH* promoters contributes to decreased *NOTCH* expression [18–20]. To further confirm the direct regulation of KMT2A in *NOTCH1* and *NOTCH3*, we used methylation-specific PCR (MSP), and the result revealed that the CpG islands in *NOTCH1* and *NOTCH3* promoters were unmethylated (Figure 4). In addition, the signaling observed for methylated *NOTCH1* and *NOTCH3* significantly increased upon KMT2A knockdown (Figure 4). This result indicates that KMT2A induces or maintains *NOTCH1* and *NOTCH3* expression by protecting *NOTCH1* and *NOTCH3* from DNA methylation.

### KMT2A upregulates NOTCH1 and NOTCH3 in U-87 MG cell proliferation

**Figure 5:**
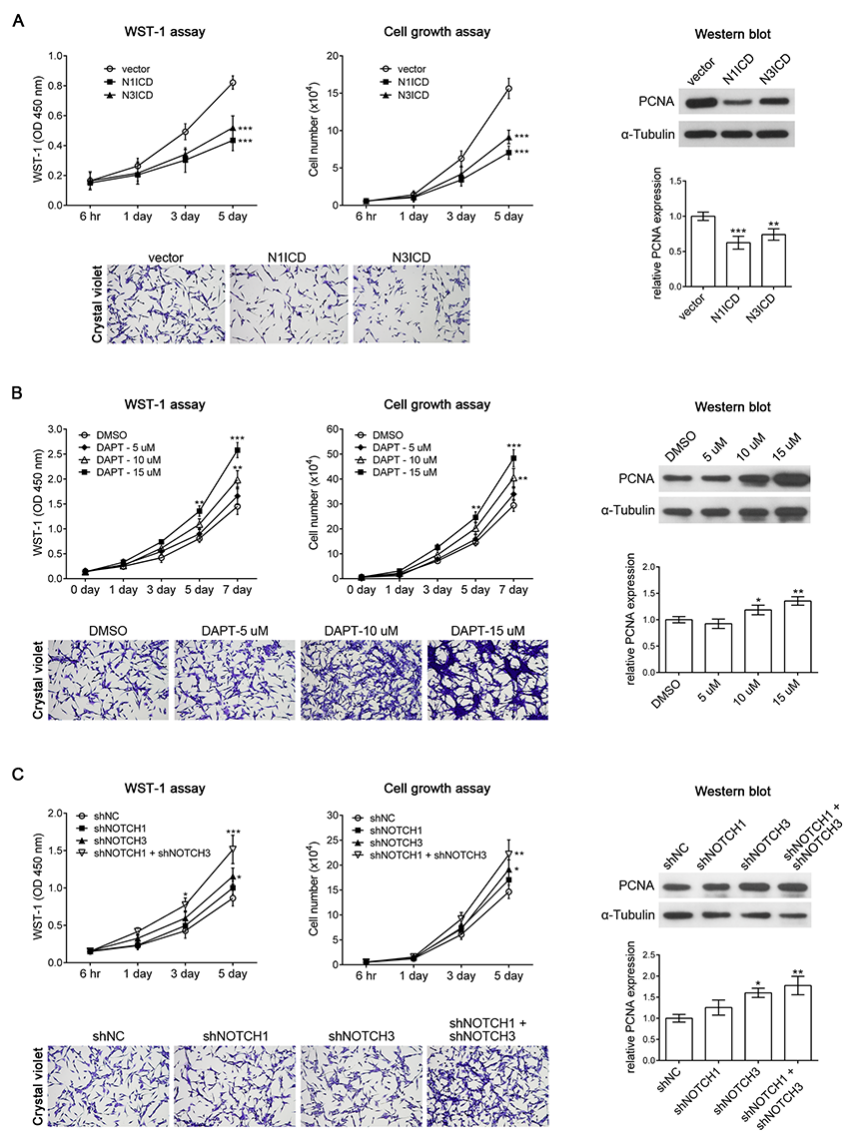
NOTCH1 and NOTCH3 suppresses U-87 MG cell proliferation **(A)** The result of the WST-1 assay, cell growth assay, and Western blot analysis with PCNA antibody revealed that overexpressing the constitutively active form of NOTCH1 (*N1ICD*) or NOTCH3 (*N3ICD*) inhibited U-87 MG cell proliferation. **(B)** U-87 MG cells treated with the NOTCH signaling inhibitor DAPT exhibited increased cell proliferation in a dose-dependent manner. DMSO was used as a control. **(C)** The WST-1 assay, cell growth assay, and Western blot analysis with PCNA antibody demonstrated that *shNOTCH1* had no effect on U-87 MG cell proliferation, *shNOTCH3* slightly induced U-87 MG cell proliferation, and double transfection of *shNOTCH1* and *shNOTCH3* exhibited significant induction of U-87 MG cell proliferation. *, p < 0.05; **, p < 0.01; ***, p < 0.001.

NOTCH1 acts as an oncoprotein in T-cell acute lymphoblastic leukemia/lymphoma. Approximately 60% of human T-cell acute lymphoblastic leukemia/lymphoma cases present NOTCH1 activation [21]. In addition, the oncogenic character of NOTCH signaling has been described in many types of tumors including breast carcinoma [22], hepatocellular carcinoma [23], pancreatic cancer [24], and brain tumors [25]. However, recent studies have revealed that NOTCH signaling can be either oncogenic or tumor suppressive, depending on the cellular context [26]. For example, NOTCH1 induces astrocytic gliomas, whereas NOTCH2 suppresses the growth of astrocytic gliomas [27]. Activation of the NOTCH pathway reduces glioma growth, and high NOTCH activity correlates with lower tumor grade and increased patient survival [28]. As mentioned, KMT2A is essential for the inhibition of cell proliferation and positively regulates NOTCH signaling, suggesting that NOTCH acts as a tumor suppressor in U-87 MG cells. We further confirmed the role of NOTCH in U-87 MG cells by overexpressing a constitutively active intracellular domain of NOTCH1 (*N1ICD*) or NOTCH3 (*N3ICD*). The results of the WST-1 assay, cell growth assay, and Western blot with PCNA antibody revealed that *N1ICD* or *N3ICD* inhibited U-87 MG cell proliferation (Figure 5A). In addition, the inhibition of NOTCH signaling by using the chemical inhibitor N-[N-(3,5-difluorophenacetyl)- L-alanyl]-S-phenylglycine t-butyl ester (DAPT) induced U-87 MG cell proliferation in a dose-dependent manner (Figure 5B). This result indicates that NOTCH activation is sufficient for inhibiting U-87 MG cell proliferation. DAPT inhibits γ-secretase activity and thus generally inhibits the activation of all NOTCH receptors [29]. Therefore, we individually inhibited *NOTCH1* and *NOTCH3* expression by using specific shRNA (*shNOTCH1* [30, 31] and *shNOTCH3* [32, 33], respectively). *shNOTCH1* did not significantly affect the proliferation of U-87 MG cells, whereas *shNOTCH3* only slightly induced the proliferation of U-87 MG cells (Figure 5C). However, double transfection of *shNOTCH1* and *shNOTCH3* significantly induced U-87 MG cell proliferation (Figure 5C). This result indicates that either NOTCH1 or NOTCH3 alone is sufficient for inhibiting U-87 MG cell proliferation, and that the double knockdown of NOTCH1 and NOTCH3 reduces the inhibition effect and induces the upregulation of U-87 MG cell proliferation.

**Figure 6:**
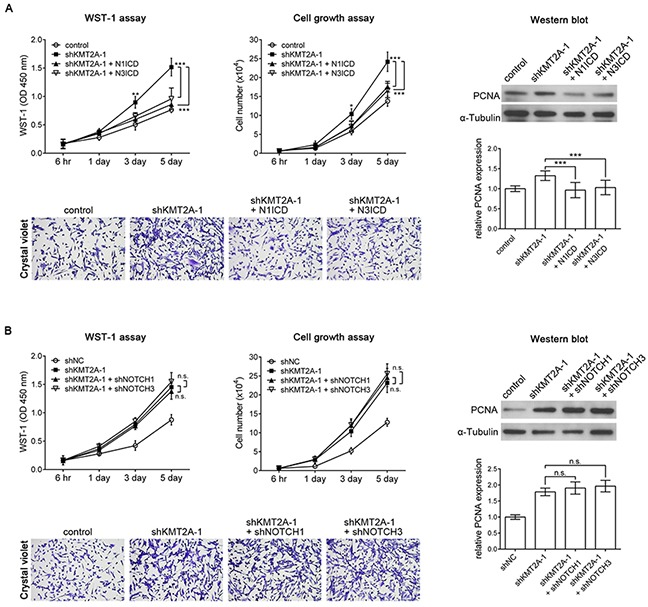
N1ICD abolishes the KMT2A knockdown effect **(A)** KMT2A knockdown-induced cell proliferation was recovered using *N1ICD* or *N3ICD*, as determined by the WST-1 assay, cell growth assay, and Western blot analysis with PCNA antibody in U-87 MG cells. **(B)** *shNOTCH1* or *shNOTCH3* in *shKMT2A*-transfected U-87 MG cells did not further induce U-87 MG cell proliferation, compared with the effect of *shKMT2A*. n.s., not significant; *, p < 0.05; **, p < 0.01; ***, p < 0.001.

According to our results, KMT2A is essential for the inhibition of U-87 MG cell proliferation through the positive regulation of NOTCH1 and NOTCH3. To further confirm this finding, we expressed *N1ICD* or *N3ICD* in KMT2A-knockdown U-87 MG cells. KMT2A knockdown induced U-87 MG cell proliferation, and this phenotype could be abolished by *N1ICD* or *N3ICD* (Figure 6A). In addition, we performed knockdown of NOTCH1 or NOTCH3 expression in U-87 MG cells with a KMT2A-knockdown background. The result showed that NOTCH1 or NOTCH3 knockdown in KMT2A-knockdown U-87 MG cells did not further induce U-87 MG cell proliferation, compared with the effect of KMT2A knockdown (Figure 6B). This result further confirmed that KMT2A-NOTCH1/3 signaling inhibits U-87 cell proliferation.

### KMT2A knockdown promotes glioma tumorigenesis *in vivo*

**Figure 7:**
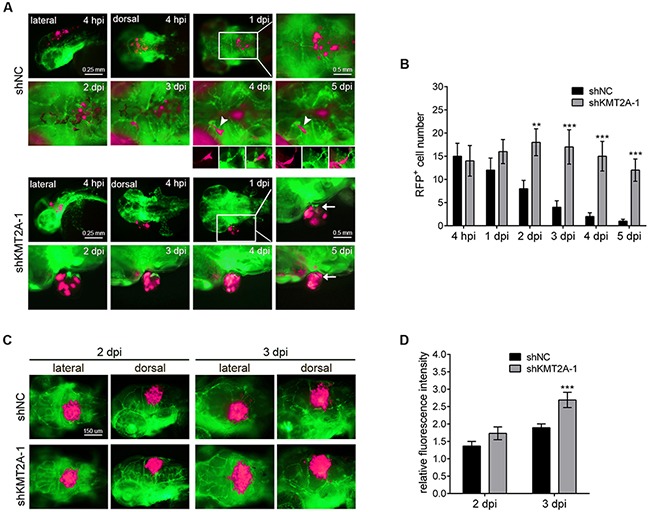
KMT2A knockdown promotes tumor growth in zebrafish **(A)** U-87 MG cells were labeled with RFP (shown in fluorescent magenta), and approximately 50 cells were injected into the brains of Tg(*fli1*:*egfp*) fish in each experiment. Blood vessels are depicted by green fluorescent color. The transplanted zebrafish were imaged from 4 hours postimplantation (hpi) to 5 days postimplantation (dpi). Arrowheads depict tumor cells in the blood vessels, and arrows indicate the zebrafish blood vessels penetrating into the tumor tissues. **(B)** Quantification of transplanted cells. Quantification was performed by individually counting cells with red fluorescence (shown in fluorescent magenta), which indicated that the number of transplanted cells was significantly increased by KMT2A knockdown, compared with the control. shNC, scramble shRNA as negative control. **(C)** The immunosuppressant dexamethasone treatment increased the tumor growth. This result was quantified by measuring the red fluorescence intensity **(D)**. **, p < 0.01; ***, p < 0.001.

Because of the advantages of rapid growth and the optical clarity of zebrafish embryos, we transplanted the KMT2A-knockdown U-87 MG cells into zebrafish brains to investigate the role of KMT2A in glioma progression *in vivo*. We used the transgenic zebrafish line with green fluorescent protein (GFP)-labeled blood vessels [Tg(*fli1*:*egfp*)] to facilitate the observation of tumor–endothelial cell interactions. The U-87 MG cells were labeled with red fluorescent protein (RFP) and implanted into the brains of 2-day postfertilization (dpf) embryos, followed by periodical examination from 4 hours to 5 days after implantation. Embryos injected with U-87 MG cells with scrambled shRNA were used as controls. In the control embryos, the transplanted U-87 MG cells gradually scattered, and the number of transplanted cells gradually decreased (Figure 7A). By contrast, the number of transplanted U-87 MG cells with KMT2A knockdown was significantly higher than the controls at all analysis stages. In particular, the transplanted tumor cells with KMT2A knockdown remained in a condensed bulk structure compared with the loose scattered cells of control embryos (Figure 7A). In addition, the transplanted tumor cells with KMT2A knockdown promoted angiogenesis by inducing zebrafish blood vessel growth into the tumor bulk (Figure 7B). This tumor angiogenesis has been demonstrated to be a critical step in localized tumor growth. To further improve the growth of the tumor graft, we treated the zebrafish with the immunosuppressant dexamethasone during transplantation [34–36]. Under the immunosuppressive condition, the U-87 MG cell mass grew in size, whereas the KMT2A knockdown further increased the size of the U-87 MG cell mass relative to the controls (Figure 7C, 7D). Therefore, the xenotransplantation result revealed KMT2A knockdown to be sufficient for promoting tumor growth and angiogenesis *in vivo*.

## DISCUSSION

Our result reveals that KMT2A negatively acts on tumor growth, in contrast to our previous understanding of KMT2A as an oncogene in tumorigenesis [13]. KMT2A rearrangements account for approximately 10% of human leukemias, and more than 70 fusion proteins have been identified [10]. Accordingly, KMT2A fusion proteins are postulated to function to induce genes of oncogenic signaling and hence induce tumor progression. However, recent studies have demonstrated that KMT2A mutations are prevalent in many tumors [37–40], because KMT2A mutations in these tumors are nonsense or frameshift mutations that lead to the early termination of translation and result in truncated proteins with no methyltransferase activity. These studies have suggested that the loss of function of KMT2A is essential for tumor progression. The close family members of KMT2A, KMT2C, and KMT2D also have negative effects on tumor cell growth [41–43], supporting the tumor-suppressive role of the KMT2 family. In conclusion, our results demonstrate that in U-87 MG cells, KMT2A positively regulates NOTCH1 and NOTCH3, and this KMT2A–NOTCH1/3 cascade is essential for the inhibition of tumor cell proliferation. This finding further substantiates the tumor-suppressive role of KMT2A in tumor progression.

Our result also demonstrates a novel regulatory mechanism in glial tumor progression. The components of the NOTCH pathway have been reported to be deregulated in numerous hematological malignancies and brain tumors. However, the role of NOTCH signaling is not consistent in these tumors. For example, in medulloblastomas, NOTCH2 was shown to promote tumorigenesis, whereas NOTCH1 inhibited tumor growth [44]. NOTCH1 expression is higher in grade II and III gliomas but lower in more malignant gliomas [45, 46]. These studies have indicated that NOTCH signaling could be oncogenic or tumor suppressive in brain tumors, depending on the signaling initiated by different NOTCH receptors and different cell types. We demonstrated that KMT2A selectively upregulates different NOTCH receptors, and this mechanism may be crucial for driving NOTCH signaling to be oncogenic or tumor suppressive. In addition, KMT2A directly acted on NOTCH1 and NOTCH3 transcription through methylation. This result highlights the KMT2A–NOTCH suppression of cell proliferation as a unique regulatory cascade in glioblastomas. Currently, many inhibitors of NOTCH signaling, such as γ-secretase inhibitors, have been tested in clinical trials based on the assumption that NOTCH signaling is oncogenic [47]. In particular, these chemical inhibitors unselectively block signals transduced by all NOTCH receptors. Therefore, we suggest that these inhibitors should be used with caution because inhibiting NOTCH signaling may reduce the tumor-suppressive character in certain types of tumors and consequently induce tumor growth.

## MATERIALS AND METHODS

### Cell culture

U-87 MG cells were purchased from the American Type Culture Collection (ATCC) and maintained in Dulbecco's modified Eagle's medium (DMEM) (Gibco, USA), containing 10% foetal bovine serum (FBS) (Gibco, USA) and antimycotic antibiotic (Gibco, USA).

### shRNA knockdown of KMT2A expression

To establish a stable shRNA-tagged RFP-expressing U-87 MG cell line, the following shRNA construct were transfected, including a non-targeting pGPU6/RFP/scramble shRNA as negative control (shNC, target sequence: 5′-TTCTCCGAACGTGTCACGT-3′) and two pGPU6/RFP/shKMT2A (*shKMT2A-1*, target sequence: 5′-GGTGTTGTCGTCGTTGCAAAT-3′; *shKMT2A-2*, target sequence: 5′-GCGCCAAGCTCTTTGCTAAAG-3′) (purchased from BioTools). Transfection was performed using DreamFect™ Gold transfection reagent (OZ Biosciences). After 8 hours, the medium was replaced with G-418 (600 μg/mL, TOKU-E) selection medium. The stable shRNA-RFP expressing U-87 MG cells were grown in DMEM culture medium containing 10% FBS, antibiotics and G-418 (300 μg/mL). The *shKMT2A-1* was more efficient in suppressing KMT2A expression in comparison to *shKMT2A-2*.

### Generation of constitutively active NOTCH1 and NOTCH3 constructs

The NOTCH1 (NM_017617) intracellular domain (N1ICD) expression plasmid was generated by PCR from human cDNA. The N1ICD fragment contains the amino acid sequences from 1761 to 2555 of NOTCH1 (GenBank: NP_060087). The transcriptional start site ATG was added within forward primer, and the ClaI restriction site was created in both forward and reverse primers to favour sequential experiments. Primer sequences used were as follow: forward primer 5′- ATCGATATGCGGCAGCATGGCCAGCTC-3′ and reverse primer 5′- ATCGATCCTTGAAGGCCTCCGGAATGCG-3′. KOD FX DNA polymerase (TOYOBO) was used for PCR. The PCR product was ClaI (Roche), which was digested and purified (Geneaid). Following cloning into ClaI linearized pCS2(+) Myc vectors, in frame with the Myc-tag, and the produce was sequenced to ensure correct cloning and sequencing.

### Real-time quantitative RT-PCR

Total RNA was isolated from cultured tumor cells using Trizol™ Reagent (Invitrogen, USA) according to the manufacturer's protocol. qRT-PCR was performed using *Power* SYBR Green PCR master mix (Applied Biosystems) on an ABI 7500 fast real-time PCR system (Applied Biosystems) according to the manufacturer's instructions. The primer sequences are listed in Supplementary Table 1. The PCR protocol included a denaturation programme (95°C for 10 min), followed by 40 cycles of amplification and quantification (95°C for 15 sec, 60°C for 1 min). Each sample was replicated at least three times.

### Genomic DNA extraction and bisulphite conversion

Genomic DNA was extracted using the standard phenol-chloroform method, and subsequently, 500 ng DNA was input for bisulphite conversion using an EZ DNA Methylation-Lightning Kit (Zymo Research) according to the manufacturer's protocol.

### Methylation specific PCR (MSP) analysis

Methylation-specific PCR (MSP) was performed for the methylation analysis of the *NOTCH1* and *NOTCH3* promoters. The primers were designed within a CpG island region. The primers used to detect the methylated reaction were *NOTCH1*-MSP forward sequence 5′-CGTGATCGTAGTTTAGTTTTTGACGT-3′ and reverse sequence 5′-ATCTCGTAAAACGCGCCGTT-3′, which amplify a 263-bp product (positions −945 to −683); and a *NOTCH3*-MSP forward sequence 5′-TGTTTAGGTTGGAGCGTAGTGGTAC-3′ and reve-rse sequence 5′-AAAAATACAAAAATTAACTAAACGTA-3′, which amplify a 142-bp product (positions −230 to −89). The primers for the unmethylated reaction were *NOTCH1*-uMSP forward sequence 5′-TGTGATTGTAGTTTAGTTTTTGATGT-3′ and reverse sequence 5′-AAATCTCATAAAACACACCATT-3′, which amplify a 265-bp product (positions −947 to −683); and *NOTCH3*-uMSP forward sequence 5′-TGTTTAGGTTGGAGTGTAGTGGTATGA-3′ and reverse sequence 5′-AAAAATACAAAAATTAACTAAACATA-3′, which amplify a 142-bp product (positions −230 to −89). The PCR results were analyzed by DNA electrophoresis.

### Western blot

Before lysis, the cells were washed once with cold phosphate-buffered saline (PBS). Total cell lysates were prepared by the addition of RIPA lysis buffer (Hycell) containing protease inhibitors (Calbiochem). Total protein was separated on bis-acrylamide gels and transferred to polyvinylidene fluoride membranes (PerkinElmer Life Sciences). Primary antibodies for KMT2A (1:500; 05-765; Millipore), PCNA (1:1000; Proteintech), or TUBULIN (1:10,000; T9026; Sigma-Aldrich) were incubated overnight at 4°C. Secondary antibodies conjugated to horseradish peroxidase (Invitrogen) were incubated for 1 hour at room temperature, protected from light.

### Chemical treatment

Notch signaling was inactivated by treating with a chemical inhibitor of γ-secretase DAPT (EMD Millipore). Briefly, DAPT was dissolved in dimethyl sulfoxide (DMSO) to prepare a stock solution (10 mM). U-87 MG cells were seeded at 3 × 10^3^ cells per well of a 96-well plate. Thereafter, the cells were treated with 200 μL DMEM medium supplemented with increasing concentrations of DAPT (1, 5, 10 and 15 μM). The control group was treated with an identical amount of DMSO.

### Detection of cell viability and proliferation by WST-1 assay

The WST-1 assays were used to determine the effect of U-87 MG cell viability after transfection or DAPT treatment. For the WST-1 assay, cells were seeded at 3×10^3^ cells/well in 96-well plates contained 200 μl cell growth medium. After the desired time point, they were then incubated with 10% WST-1 reagent (#630118; Clontech) for 30-90 minutes, and the absorbance was measured at 450 nm using a Multiskan FC Microplate Photometer (Thermo Fisher Scientific).

### Cell growth assay

For the cell growth assay, 5000 cells/well were seeded in 24-well plates in triplicate and allowed to grow for 6 hours or 1, 3, or 5 days. At each time point, the cells were trypsinized and the numbers of cells were directly counted using a hemocytometer. Concurrently, 10 000 cells/well were seeded in 6-well plates in duplicate. After growth for 5 days, the wells were rinsed twice with PBS, fixed for 10 minutes with cold absolute methanol in a freezer, and stained with 0.5% crystal violet (Sigma-Aldrich) in 25% methanol for 10 minutes. The stained cells were washed with ddH2O and allowed to air-dry, and images were captured with a 20× objective lens.

### Zebrafish xenotransplantation

All experiments were performed in strict accordance with standard guidelines for zebrafish studies and approved by the Institutional Animal Care and Use Committee of Chang Gung University (IACUC approval number: CGU12–039). For zebrafish xenotransplantation, Tg(*fli1:EGFP*) strains of transgenic zebrafish embryos were maintained in 0.3% phenylthiourea (PTU, Sigma-Aldrich) after 24 hours post-fertilization (hpf). At 48 hpf, these embryos were dechorionated and anaesthetized in E3 buffer contained 0.3% phenylthiourea and 0.04 mg/mL tricaine (Sigma-Aldrich) before human cell injection. The RFP-expressing U-87 MG cells were washed and re-suspended in Hank's balanced salt solution (HBSS). Approximately 15 glioma-RFP cells were injected into the hindbrain ventricles of each embryo. After confirmation of proper visible tumor cells at the injection site, zebrafish were transferred individually into 24-well dishes and maintained in E3 buffer containing PTU at 34°C. Later, individual recipients were imaged for tumor growth under a fluorescence microscope (Leica). Dexamethasone was used as an immunosuppressant to improve the growth of the tumor graft. Larval fish of 48 hpf age were treated in 100 μg/mL of dexamethasone before transplantation [34–36]. The transplanted cells were quantified by counting the RFP-positive cells individually or measuring the relative fluorescence intensity of the RFP-labeled cells. Knockdown of KMT2A by monitored by detecting the C-terminal p180 (*MLL^C^*) fragments by using qRT-PCR at different time points in the zebrafish transplanted with tumor cells (supplementary Figure 1).

### Statistical analysis

Quantitative data are presented as the means ± SD determined from the indicated number of experiments. The statistical analysis was based on Student's t-tests for comparing two groups or one-way ANOVA for multiple comparisons. P values lower than 0.05 was considered statistically significant.

## SUPPLEMENTARY MATERIALS FIGURES AND TABLE




