# Supplementary material for: Epigenetic regulation of NOTCH1 and NOTCH3 by KMT2A inhibits glioma proliferation
Source: Oncotarget. 2017 Jun 27;8(38):63110–20. doi: 10.18632/oncotarget.18668 (PMC5609907; doi:10.18632/oncotarget.18668)
Supplement: Supplementary file 1 [file oncotarget-08-63110-s001.pdf]

Epigenetic regulation of NOTCH1 and NOTCH3 by KMT2A inhibits glioma proliferation

SUPPLEMENTARY MATERIALS

SUPPLEMENTARY FIGURES AND TABLE

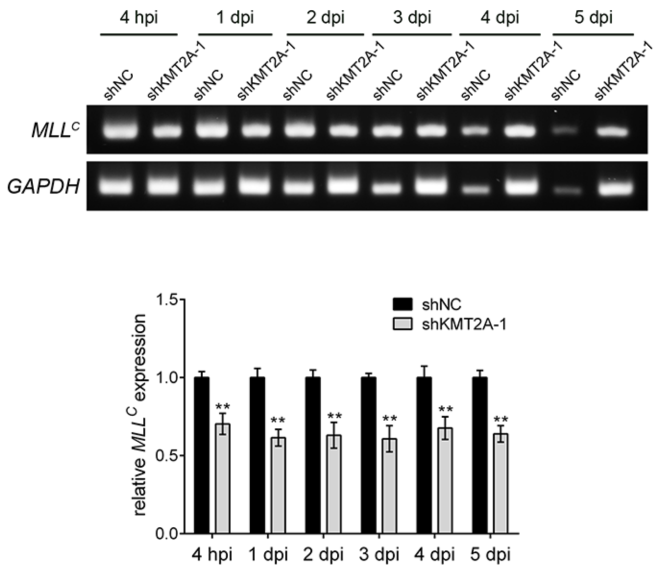

Supplementary Figure 1: *shKMT2A* was effective at all analysis stages. The effectiveness of *shKMT2A* was examined by detecting the expression of MLL^C fragments by using qRT-PCR at different time points, and the result revealed that *shKMT2A* was functional and sufficient for inhibiting *KMT2A* expression at all analysis stages. In the controls (shNC), the gradually decreased expression of MLL^C was due to the reduced size of the tumor graft. Therefore, quantification was performed by normalizing the expression of MLL^C in the *shKMT2A*-transfected sample to the *GAPDH* expression level and then normalizing to the expression of MLL^C in the controls.

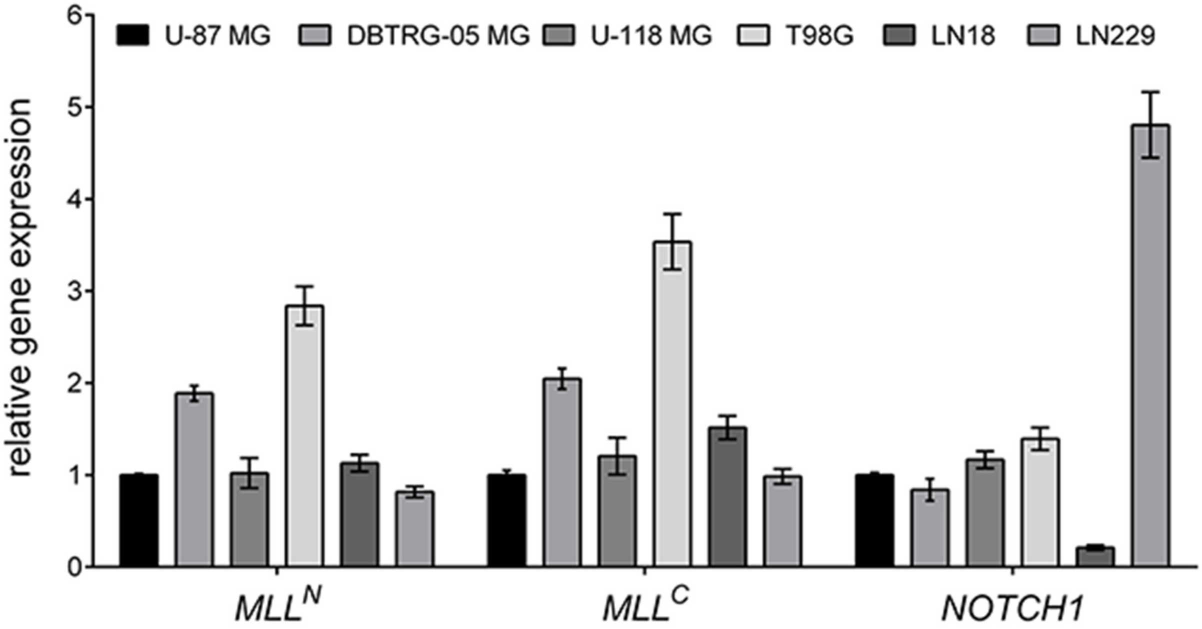

Supplementary Figure. 2: Different expression levels of KMT2A and NOTCH1 in brain tumor cells. RT-PCR analysis showing MLLN, MLLC, and NOTCH1 are expressed in different levels in different brain tumor cells.

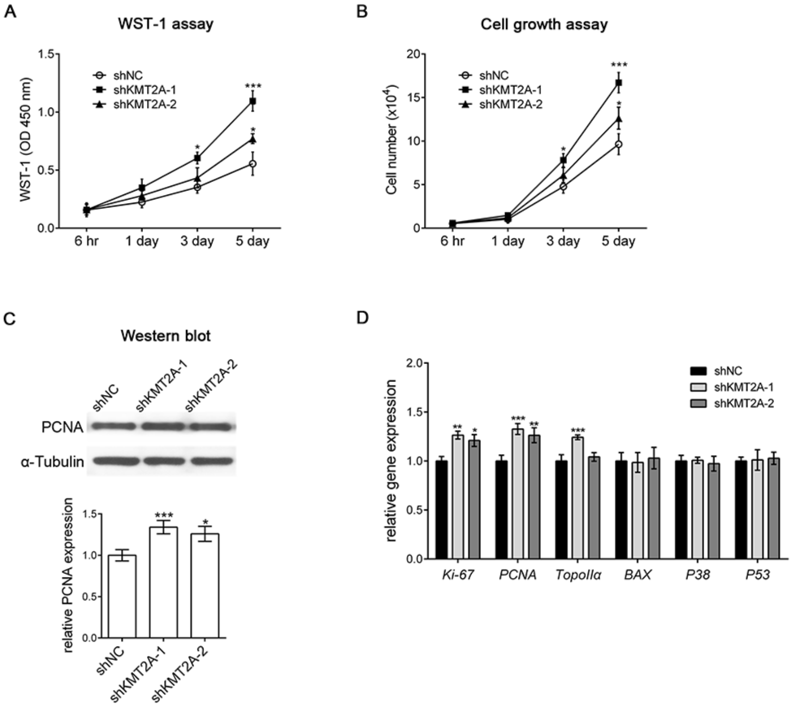

Supplementary Figure. 3: shKMT2A induces U-118 MG cell proliferation. WST-1 assay **(A)**, cell growth assay **(B)**, and Western blot analysis with PCNA antibody **(C)** demonstrated that shKMT2A induces U-118 MG cell proliferation. **(D)** Cell proliferation and survival markers were analyzed using real-time RT-PCR, showing that shKMT2A induces proliferation markers [Ki-67, PCNA, and Topoisomerase IIα (TopoIIα)] without significant alteration of apoptosis markers (BAX, P38 MAPK, and P53). (E) Representative images revealing that shKMT2A induces the growth of U-118 MG cells. Cells were stained with crystal violet. *, $p < 0.05$; **, $p < 0.01$; ***, $p < 0.001$.

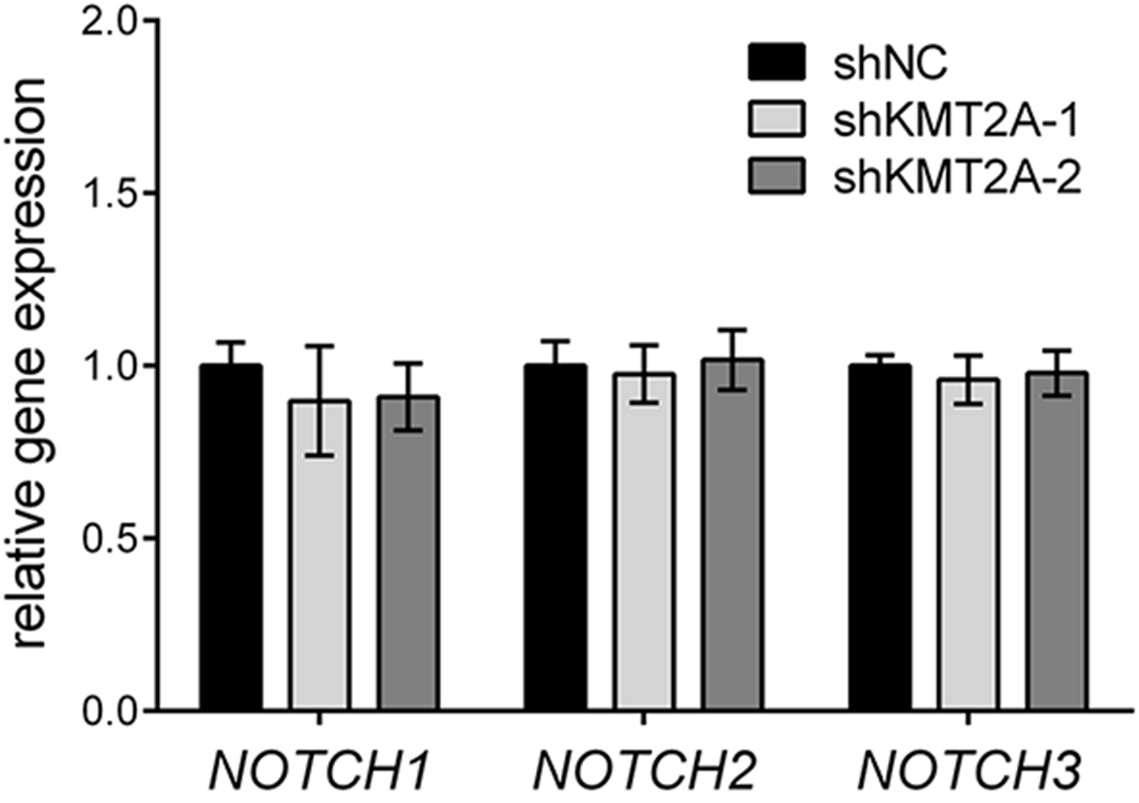

Supplementary Figure. 4: KMT2A knockdown does not affect NOTCH expression. The expression of NOTCH was examined using real-time PCR. In KMT2A-deficient U-118 MG cells, the expression levels of NOTCH1, NOTCH2, and NOTCH3 were not altered by KMT2A shRNAs .

Supplementary Table 1: The complete list of primer sequences used in this study.

See Supplementary File 1
