# Supplementary material for: Epigenetic regulation of NOTCH1 and NOTCH3 by KMT2A inhibits glioma proliferation
Source: Oncotarget. 2017 Jun 27;8(38):63110–20. doi: 10.18632/oncotarget.18668 (PMC5609907; doi:10.18632/oncotarget.18668)
Supplement: Supplementary file 2 [file oncotarget-08-63110-s002.doc]

**Supplementary Table 1**

**The complete list of primer sequences used in this study.**

| Gene symbol | Accession number |  | primers sequence (5' to 3') |
| --- | --- | --- | --- |
| *KMT2A^N^* | NM_001197104 | Forward | CCCCTTCGGGTCTCCTCATT |
|  |  | Reverse | ATCCTTCGAGGGCTTTGTCTG |
| *KMT2A^C^* | NM_001197104 | Forward | CAGATAAAGTCCAGGAAGCTCG |
|  |  | Reverse | GTAATTTCGACAGTGCTTGGC |
| *Ki-67* | NM_002417 | Forward | gaggtgtgcagaaaatccaaa |
|  |  | Reverse | ctgtccctatgacttctggttgt |
| *PCNA* | NM_002592 | Forward | TTACTGAGGGCGAGAAGCG |
|  |  | Reverse | AGTCTAGCTGGTTTCGGCTT |
| *TopoIIα* | NM_001067 | Forward | cagtgaagaagacagcagcaa |
|  |  | Reverse | agctggatcccttttagttcct |
| *TPX2* | NM_012112 | Forward | acatctgaactacgaaagcatcc |
|  |  | Reverse | ggcttaacaatggtacatccctta |
| *BCL-2* | NM_000633 | Forward | AGGAAGTGAACATTTCGGTGAC |
|  |  | Reverse | GCTCAGTTCCAGGACCAGGC |
| *BAX* | NM_001291428 | Forward | TGCTTCAGGGTTTCATCCAG |
|  |  | Reverse | GGCGGCAATCATCCTCTG |
| *P38MAPK* | NM_001315 | Forward | GCCCAAGCCCTTGCACAT |
|  |  | Reverse | TGGTGGCACAAAGCTGATGAC |
| *TP53* | NM_000546 | Forward | CTGGCCCCTGTCATCTTCTG |
|  |  | Reverse | CCGTCATGTGCTGTGACTGC |
| *NOTCH1* | NM_017617 | Forward | ACCAATACAACCCTCTGCGG |
|  |  | Reverse | GGCCCTGGTAGCTCATCATC |
| *NOTCH2* | NM_024408 | Forward | CAACCGCCAGTGTGTTCAAG |
|  |  | Reverse | GAGCCATGCTTACGCTTTCG |
| *NOTCH3* | NM_000435 | Forward | CGTCAGTGTGAACTCCTCTC |
|  |  | Reverse | GGTTGGTGCAGATACCATGAG |
| *HES1* | NM_005524 | Forward | AAGAAAGATAGCTCGCGGCA |
|  |  | Reverse | TACTTCCCCAGCACACTTGG |
| *HES3* | NM_001024598 | Forward | GAAAGTCTCCCTGGCTCGTC |
|  |  | Reverse | CCAAATAGGGAGCGCCTTCA |
| *HES5* | NM_001010926 | Forward | GAAAAACCGACTGCGGAAGC |
|  |  | Reverse | GACGAAGGCTTTGCTGTGCT |
| *TBP* | NM_003194 | Forward | GAGCTGTGATGTGAAGTTTCC |
|  |  | Reverse | TCTGGGTTTGATCATTCTGTAG |
